# Social support, emotion regulation and mindfulness: A linkage towards social anxiety among adolescents attending secondary schools in Birgunj, Nepal

**DOI:** 10.1371/journal.pone.0230991

**Published:** 2020-04-02

**Authors:** Rakesh Singh, Babita Singh, Sharika Mahato, Victoria K. Hambour

**Affiliations:** 1 School of Public Health, Patan Academy of Health Sciences, Lalitpur, Nepal; 2 National Medical College and Teaching Hospital, Tribhuvan University, Birgunj, Nepal; 3 The Hebrew University-Hadassah Braun School of Public Health and Community Medicine, Jerusalem, Israel; 4 School of Applied Psychology, Griffith University, Mount Gravatt, Australia; University of York, UNITED KINGDOM

## Abstract

There has been a growing burden of anxiety among Nepalese adolescents. Social anxiety in particular is one of the commonly reported symptoms indicating mental health problem among adolescents. The purpose of this study was to assess social anxiety, and identify how social support, emotion regulation and mindfulness uniquely contribute to social anxiety among adolescents in Birgunj, Nepal. The study was conducted by using a self-administered questionnaire among 384 adolescents (65.4% boys; M = 16.05 years, SD = 1.39) studying at secondary schools of Birgunj. Results show that there was a positive correlation between social anxiety symptoms and age, and girls reported more symptoms. Traits such as non-acceptance of emotions, lack of clarity and lack of awareness of emotions were related to increased social anxiety; while acting with awareness, non-reactivity, and better ability to describe emotions was related to decreased social anxiety. Finally, more social support from close friends was related to lower social anxiety. These results suggest that improving emotion regulation, dispositional mindfulness, and social support may be helpful for adolescents who are at risk of, or are suffering from, social anxiety.

## 1. Introduction

Mental health is an important contributor to better quality of life, and low anxiety is a key indicator of mental health. The American Psychological Association defines anxiety as “an emotion characterized by feelings of tension, worried thoughts and physical changes like increased blood pressure” [1]. Anxiety consists of bodily symptoms, a cognitive appraisal and most often a tendency to behave in a certain way (e.g., avoidance).

Anxiety lies among the most common mental illnesses with a lifetime prevalence of more than 20% [2]. Social anxiety disorder is one of the most common types of anxiety disorder with the feelings of anxiety, fear, and uneasiness in social situations such as meeting new people and answering a question in a class [3,4]. Social anxiety is a symptom or even a normative phenomenon where an individual is in the fear of social situations involving interaction with people [3,4]. Thus, social anxiety is a symptom whereas anxiety disorder is clinical. Previous studies have indicated that people with social anxiety are characterized by decreased ability to control and manage their emotions [5,6]. Emotion dysregulation in the context of social anxiety is the inability of an individual to influence or control their own emotional experiences [7]. Mindfulness has been described as the ability to non-judgmentally identify and interpret one’s experiences including emotional experience [8]; the extent to which one innately engages in these behaviours without formal training is known as dispositional mindfulness [9].

Social anxiety generally starts during adolescence and can persist for years, or even a lifetime, if not identified and treated [10]. Adolescence is a crucial stage for all round development to healthy adulthood. If social anxiety is not managed or treated, it can prevent sufferers from reaching their full potential [3].

Social anxiety is linked with deterioration in adolescents’ functioning including: hampered social and personal relationships, school absenteeism or dropout, depression, suicide, substance abuse, and increased behavioral problems such as fighting, and cheating [11–15].

The mental health of adolescents is a major concern worldwide, including low-income countries like Nepal where there is low priority towards both mental health and adolescents [16,17]. A national survey conducted among adolescents in Nepal showed that three quarters suffered from anxiety and more than two fifths of them reported feelings of hopelessness [18].

A study conducted in the western region of Nepal showed that adolescent girls had higher levels of anxiety than boys [19]. Conversely, a second study conducted in the same region where 46.5% of the total sample reported anxiety symptoms, boys reported more symptoms than girls [20]. Overall, it is clear that anxiety is a widespread concern for adolescents in these regions.

Although social anxiety has been identified long ago as a significant indicator of adult’s functioning [21], there is paucity in research to assess social anxiety among Nepalese adolescents. Social support towards adolescents provided by the societal members such as family, relatives, friends, peers, and teachers play a vital role to develop necessary social skills for proper adult functioning [22,23]. Given the negative influence of social anxiety during adolescence, and the potential flow-on effect for mental health in adulthood, social anxiety is an important condition requiring further investigation in the Nepalese context.

In a study to assess the constructs of social anxiety and to determine the unique associations of social anxiety with emotion dysregulation and mindfulness among the sample of Australian adolescents, it was found that the emotion dysregulation subscale including limited strategies, and the mindfulness subscales including observing and describing were having unique association with social anxiety [5]. Moreover, while strategies and observing was found to have associated with higher symptoms of social anxiety, describing was associated with lower symptoms [5].

Although studies in the past have explored the constructs of social anxiety among adolescents, none have concentrated on Nepalese adolescent’s sample. So, this study attempted to fill the gap of assessing the constructs of social anxiety among Nepalese adolescents. Furthermore, compared to the Australian study [5], with the addition of social support as a construct of social anxiety, the current study aimed to assess social anxiety, and test the unique associations of social support, emotion regulation, and dispositional mindfulness with social anxiety among Nepalese adolescents enrolled in secondary schools in Birgunj, Nepal.

## 2. Materials and methods

### 2.1 Study design, participants and procedures

This cross-sectional study was carried out in August and September 2018 among adolescents from grades 9 to 12 and aged between 13 and 19 years, attending secondary schools of Birgunj, Nepal. Students with characteristics of difficulty in listening or with reported severe mental illness were the exclusion criteria for the study. Data was collected from 384 adolescents (65.4% boys)with mean age of 16.05 years (SD = 1.39).Students from four secondary schools (two public and two private) completed filling self-administered questionnaire during normal classroom time in presence of the researchers. The schools were selected by simple random sampling from the list of available secondary schools in Birgunj and 26 students from each stratum (grade- 9, 10, 11 and 12) were randomly picked by lottery method.

Ethical clearance was obtained from Institutional Review Committee of National Medical College (FNMC-311-074-075). First, the purpose of the study was explained to the four randomly selected schools’ principal, and their permission to conduct research with school students was sought, with all principals providing consent. Second, as parental consent was needed for student participation, it was requested in a letter sent home with the students. After two days, all the letters were obtained with signed parental consent to participate in the study. Third, the study purpose was explained to the selected students in a classroom setting in their respective school, followed by the process of seeking written assent from them prior to administration of the questionnaire. All of the selected students provided their assent to participate in the study. Further, the student participants were informed of their right to refuse to participate, and were assured that confidentiality and anonymity would be maintained at all stages of the study.

### 2.2 Data collection tool

Data was collected using a structured questionnaire consisting of five sections. Section one contained questions assessing socio-demographic characteristics. Section two assessed social anxiety using the 18-item Social Anxiety Scale for Adolescents (SAS-A) [4].Section three utiliseda5-item scale to measure self reported social support from different sources including classmates, close friends, parents, relatives and teachers. Section four contained the 18-item Difficulties in Emotion Regulation Scale (DERS-18) [24] to measure emotion dysregulation. Finally, section five assessed dispositional mindfulness with the 39-item Five Factor Mindfulness Questionnaire (FFMQ) [9].

The SAS-A is comprised of three subscales [4] including Fear of Negative Evaluation (e.g. “I worry about being teased”), where increased scores were indicative of the person experiencing increased anxiety in situations in which they are likely to be evaluated by others. Two other subscales assess Social Avoidance and Distress, with higher scores indicating increased anxiety in new situations (e.g. “I get nervous when I meet new people”), and in more general situations (e.g. “I feel shy even with peers I know very well”). In addition to the individual subscale scores, averaging all items provides a total social anxiety score, this was the score utilised in the present study. Total scores can range between 1 and 5 with a rating of 5 indicating higher levels of anxiety. The DERS-18 assesses six facets of emotion dysregulation including non-acceptance of emotional response (non-accept), impulse control difficulties (impulse), limited access to strategies (limited strategies), difficulties engaging in goal oriented behaviour (goal difficulties), lack of emotional clarity (lack of clarity), and lack of emotional awareness (lack of awareness) [24]. Items were rated on a 5-point Likert-type scale. Several of the items were reverse-scored. Following this, relevant items were averaged to form the subscale scores. Averaging all of the items provided an overall measure of emotion dysregulation. Scores for the overall measure as well as all subscales can range from 1 to 5, with higher scores indicating greater emotion regulation difficulties. Five subscales of dispositional mindfulness comprise the FFMQ [9] including Acting with Awareness (Act Aware; e.g “I am easily distracted”) which assesses the ability to remain consciously engaged with a task or experience. Observing (e.g. “I notice the smells and aromas of things”) is the second subscale; it reflects the tendency to consciously notice experiences and sensations. The third subscale, describing (e.g. “My natural tendency is to put my experiences into words”), indicates one’s ability to put experiences into words. The fourth subscale, Nonjudging of Experience (Nonjudge; e.g. “I disapprove of myself when I have irrational ideas”) represents the tendency to be comfortable with and accepting of one’s negative emotions. Finally, Nonreactivity to Inner Experience (Nonreact; e.g. “I watch my feelings without getting lost in them”) relates to one’s tendency to notice inner experiences, particularly distressing ones, without immediately reacting. All subscales contain eight items except for Nonreact, which contains seven items. Response options for the FFMQ were on a 5-point Likert-type scale where higher scores indicated increased dispositional mindfulness for all subscales. Several items were reverse-scored; following this averaging the appropriate items provided subscale scores. Responses for all scales ranged from 1 (Never) to 5 (Always).

The original English language questionnaire was translated into Nepali, followed by a back-translation to English by a researcher who was blind to the original questionnaire. The three versions of the questionnaire were then reviewed by assessing the meaning of each word to ensure the accuracy of the translation. The final Nepali language questionnaire was pilot testedwith a sample of 39 Nepalese adolescents studying at secondary school in Birgunj. The analysis of the pilot data showed that the questionnaire was reliable in the Nepalese context with Cronbach’s alpha for all measures being adequate (SAS-A = 0.849, social support scale = 0.708, DERS-18 = 0.740, and FFMQ = 0.844).

### 2.3 Overview of data analyses

Data was analyzed in SPSS vs25. Descriptive statistics such as mean and standard deviation were used to describe social anxiety, social support, emotion dysregulation and dispositional mindfulness. Total scores and subscale scores for each of the primary measures (social anxiety, social support, emotional dysregulation and dispositional mindfulness) were computed by first reverse-scoring appropriate items, then averaging the relevant items for each subscale or total score. Social anxiety as the criterion variable, to examine the associations, correlations was examined first. Bonferroni adjustment of significance levels was applied for multiple comparisons/multiple correlation analysis (Bonferroni-corrected significance level: 0.05/18 = 0.0027). Then regression models were fit to test the unique associations of social support, emotion dysregulation and dispositional mindfulness with social anxiety. Age and sex were included as control variables in the regression models.

## 3. Results

### 3.1 Correlations between social anxiety and; age, emotion dysregulation, dispositional mindfulness, and social support

Means and standard deviations for all variables included in the regression analyses can be found in Table 1. The findings reveal that social anxiety was positively correlated with two of the emotion dysregulation subscales (non-accept and lack of clarity) and negatively correlated with lack of awareness. Similarly, social anxiety was negatively correlated with two dispositional mindfulness subscales (describing and act aware) and negatively correlated with total social support score and three subscales/sources of social support (friends, relatives and teachers).

**Table 1:** Descriptive statistics and correlations between social anxiety and; subscales of emotion dysregulation, subscales of dispositional mindfulness, subscales of social support, and age; N = 384.

	1.	2.	3.	4.	5.	6.	7.	8.	9.	10.	11.	12.	13.	14.	15.	16.	17.	18.	19.
1. Age	-	.09	-.07	-.15	-.11	.07	.21**	.20**	.02	.04	.18**	.02	-.28**	.28**	.10	-.17*	- .04	-.27**	-.04
2. Social Anxiety		-	-.22**	.36**	.39**	.02	.01	.10	.001	-.24**	.04	-.08	-.37**	-.06	-.16	-.06	-.21**	-.21**	-.25**
3. ED Awareness			-	-.25**	-.10	-.28**	-.12	-.13	-.31**	-.21**	.13	-.14	.14	-.05	-.16*	-.11	-.03	.01	-.10
4. ED Clarity				-	.22**	-.03	.02	.05	.15	-.27**	.03	.04	-.29**	-.05	-.15	.05	-.12	-.04	-.11
5. ED Nonaccept					-	.11	.05	.16	.03	-.13	-.14	.06	-.26**	-.02	.002	-.01	-.13	-.07	-.09
6. ED Goals						-	.40**	.15	.14	.13	-.20**	.23**	-.20**	.06	.09	.05	-.05	.002	.04
7. ED Impulse							-	.33**	.11	.06	-.07	.10	-.31**	.20**	.08	-.01	-.07	-.10	.03
8. ED Strategies								-	.24**	.10	-.18*	.06	-.18*	.23**	.06	-.02	.06	-.01	.11
9. DM Observing									-	.31**	-.44**	.26**	-.10	-.03	.06	.11	.15	-.03	.07
10. DM Describing										-	-.13	.13	.22**	.01	-.03	.23**	.28**	.09	.18**
11. DM Nonjudge											-	-.47**	.09	-.01	-.19**	-.20**	-.08	-.24**	-.23**
12. DM Nonreact												-	-.15	.12	.11	.001	.04	.18**	.16*
13. DM Actaware													-	-.06	.01	.15	.13	.28**	.18*
14. SS Classmates														-	.40**	.15	.22**	.07	.61**
15. SS Friends															-	.27**	.10	.11	.58**
16. SS Parents																-	.22**	.23**	.51**
17. SS Relatives																	-	.33**	.66**
18. SS Teachers																		-	.64**
19. SS Total																			-
Mean	16.05	2.31	2.94	2.45	2.47	3.04	2.56	2.58	3.03	3.14	2.95	2.81	3.22	4.05	4.46	4.78	3.63	3.76	4.13
SD	1.39	0.63	0.86	0.86	0.98	1.09	1.05	0.89	0.70	0.53	0.69	0.60	0.61	1.08	0.92	0.60	1.15	1.30	0.62

Bonferroni-corrected sig. level = .05/18 = 0.0027

* p< .0027

** p < .001, ED = Emotional Dysregulation, DM = Dispositional Mindfulness, SS = Social Support

### 3.2 Associations of emotion dysregulation, dispositional mindfulness and social support with social anxiety

Five regression models were tested to examine the unique associations of social anxiety with a) the subscales of emotion dysregulation, b) the subscales of dispositional mindfulness, c) 11 subscales of emotion dysregulation and dispositional mindfulness d) the subscales of social support, e) all 16 subscales together (Table 2). When age and sex were entered as controls, they were associated with a significant amount of the variance in social anxiety. Combined they explained 5.7% of this variance, when examined individually; they were both significant predictors of social anxiety, with sex being the stronger predictor.

**Table 2:** Regression analysis of social anxiety on sex, age, emotional dysregulation subscales, dispositional mindfulness subscales and social support subscales; N = 384.

Variables	B	SE (B)	95% Confidence Intervals (B)		β
			Lower	Upper	
Emotion Dysregulation model, F(6, 375) = 21.09, p<0.001; R^2^ = 0.31					
Sex	0.242	0.058	0.128	0.357	0.183**
Age	0.092	0.021	0.051	0.133	0.204**
DERS lack of awareness	-0.083	0.034	-0.150	-0.015	-0.112*
DERS lack of clarity	0.192	0.034	0.125	0.259	0.262**
DERS nonacceptance	0.222	0.029	0.165	0.278	0.346**
DERS goal difficulties	-0.026	0.028	-0.082	0.029	-0.045
DERS Impulse	-0.031	0.030	-0.090	0.027	-0.052
DERS limited strategies	0.003	0.033	-0.062	0.068	0.005
Dispositional Mindfulness model, F(5, 376) = 15.01, p<0.001, R^2^ = 0.218					
Sex	0.275	0.062	0.154	0.397	0.208**
Age	0.017	0.023	-0.027	0.062	0.038
DM observing	0.044	0.048	-0.050	0.139	0.049
DM describing	-0.193	0.060	-0.310	-0.075	-0.162**
DM nonjudge	0.021	0.052	-0.081	0.123	0.023
DM nonreactivity	-0.144	0.056	-0.253	-0.034	-0.136*
DM acting with awareness	-0.342	0.053	-0.445	-0.239	-0.33**
Emotion Dysregulation and Dispositional Mindfulness together model, F(11, 370) = 17.5, p<0.001, R^2^ = 0.381					
Sex	.252	.056	.143	.362	.191**
Age	.061	.022	.018	.104	.135**
DERS lack of awareness	-.125	.035	-.193	-.057	-.17**
DERS lack of clarity	.124	.036	.052	.195	.169**
DERS nonacceptance	.189	.028	.133	.245	.294**
DERS goal difficulties	-.017	.028	-.071	.037	-.030
DERS Impulse	-.054	.029	-.111	.003	-.091
DERS limited strategies	.020	.033	-.044	.084	.028
DM observing	-.035	.046	-.125	.055	-.039
DM describing	-.136	.058	-.249	-.023	-.114*
DM nonjudge	.008	.048	-.087	.102	.008
DM nonreactivity	-.150	.051	-.249	-.050	-.142**
DM acting with awareness	-.212	.052	-.314	-.109	-.204**
Social Support model, F(5, 376) = 7.398, p<0.001, R^2^ = 0.121					
Sex	0.243	0.067	0.112	0.374	0.184**
Age	0.043	0.024	-0.005	0.091	0.096
SS classmates	0.008	0.033	-0.057	0.072	0.013
SS close friends	-0.096	0.038	-0.169	-0.022	-0.139*
SS parents	0.055	0.055	-0.053	0.163	0.053
SS relatives	-0.065	0.030	-0.123	-0.006	-0.118*
SS teachers	-0.073	0.026	-0.125	-0.022	-0.15**
Full model including Emotion Dysregulation, Dispositional Mindfulness and Social Support; F(16, 365) = 13.961, p<0.001, R^2^ = 0.408					
Sex	.223	.057	.112	.334	.169**
Age	.063	.023	.019	.108	.140**
DERS lack of awareness	-.153	.035	-.221	-.084	-.207**
DERS lack of clarity	.094	.037	.020	.167	.128*
DERS nonacceptance	.184	.028	.128	.240	.287**
DERS goal difficulties	-.020	.027	-.074	.033	-.035
DERS Impulse	-.051	.029	-.108	.006	-.085
DERS limited strategies	.029	.033	-.036	.093	.041
DM observing	-.043	.047	-.135	.048	-.048
DM describing	-.179	.061	-.298	-.059	-.15**
DM nonjudge	-.019	.050	-.117	.079	-.021
DM nonreactivity	-.121	.051	-.222	-.020	-.115*
DM acting with awareness	-.187	.053	-.291	-.082	-.18**
SS classmates	-.004	.029	-.060	.052	-.007
SS close friends	-.108	.033	-.174	-.042	-.158**
SS parents	.069	.049	-.026	.165	.067
SS relatives	-.018	.026	-.069	.034	-.032
SS teachers	-.034	.024	-.080	.013	-.069

*p<0.05

**p<0.01

The emotion dysregulation model explained 31.0% of the variance in social anxiety (Table 2). Sex and age remained significant predictors. The following facets of emotion dysregulation were significant predictors of social anxiety, in order from strongest to weakest according to the beta weights: non-acceptance of emotional responses (.346), lack of clarity (.262), age (.204), sex (.183), lack of emotional awareness (-.112).

The dispositional mindfulness model explained 21.8% of the variance in social anxiety (Table 2). Sex was still a significant predictor; however age was no longer significant. The following facets of dispositional mindfulness were significant predictors of social anxiety, in order from strongest to weakest according to the beta weights: acting with awareness (-.330), sex (.208), describing (-.162), non-reactivity to inner experience (-.136).

The emotion dysregulation and dispositional mindfulness combined model explained 38.1% of the variance in social anxiety (Table 2). Sex and age were both still significant predictors. The following facets of emotion dysregulation and dispositional mindfulness were significant predictors of social anxiety, in order from strongest to weakest according to the beta weights: non-acceptance of emotional responses (.294), acting with awareness (-.204), sex (.191), lack of emotional awareness (-.170), lack of clarity (.169), non-reactivity to inner experience (-.142), age (.135), describing (-.114).

The social support model explained 12.1% of the variance in social anxiety (Table 2). Sex was still a significant predictor; however age was no longer significant. The following types of social support were significant predictors of social anxiety, in order from strongest to weakest according to the beta weights: sex (.184), close friends (-.139), teachers (-.150), relatives (-.118).

In the full model, 40.8% of the variance in social anxiety was explained by the combination of age, sex, and subscales of social support, emotion dysregulation and dispositional mindfulness (Table 2). The following facets of emotion dysregulation, dispositional mindfulness, and social support were significant predictors of social anxiety, in order from strongest to weakest according to the beta weights: non-acceptance of emotional responses (.287),lack of emotional awareness (-.207), acting with awareness (-.180), sex (.169), close friends (-.158), describing (-.150),age (.140), lack of clarity (.128), non-reactivity to inner experience (-.115).

## 4. Discussion

The overall findings shows that mean social anxiety score among Nepalese adolescent students in the current study were 2.31. This result is similar to that of Australian study where the mean social anxiety score was 2.22 [5]. When examined individually, sex and age both were found to be significant predictors of social anxiety, with females and older adolescent reporting more symptoms. Anxiety thoughts including social anxiety do affect girls more than boys; they have more metacognitive beliefs about uncontrollability of worry and believe that worry must be avoided [25]. Furthermore, perhaps due to cultural and psychological influences, girls may be more likely to report their symptoms, boys may rise to believe more in their personal control over the situation; boys may experience more social pressure than girls to face fears [26]. Social support was also a significant predictor of social anxiety. When social support from close friends, relatives, and teachers were higher, adolescents reported lower symptoms.

In the current study, when emotion dysregulation and dispositional mindfulness together were regressed onto social anxiety, correlations with age and sex remained significant. Furthermore, higher reports of non-acceptance of emotions, lack of clarity and having less difficulty with emotional awareness were associated with increased social anxiety. Likewise, reports of increased acting with awareness, not reacting to inner experience, and being able to describe inner experience were associated with lower social anxiety. The Australian study also showed that sex remained associated with social anxiety, with adolescent girls reporting higher social anxiety than boys [5]. The model in Australian adolescents explained that 45% of the variance in social anxiety was accounted for age, sex, subscales of emotion dysregulation and dispositional mindfulness. In the Australian sample, limited access to emotion regulation strategies, and the tendency to observe experiences and sensations consciously, was associated with higher social anxiety [5].

When emotion dysregulation, dispositional mindfulness, and social support combined were regressed onto social anxiety, correlations with age and sex remained significant. Higher reports of non-acceptance of emotions and lack of clarity were associated with increased social anxiety, as well as having less difficulty with emotional awareness. Reports of increased acting with awareness, not reacting to inner experience, and being able to describe inner experience were associated with lower social anxiety, as was more social support from close friends. Similar findings were reported in studies among adults and Australian high school students which revealed emotion dysregulation and dispositional mindfulness were found to be associated with social anxiety [27–29].

The results of the current study indicate that there are unique associations of emotion regulation, dispositional mindfulness, and social support with adolescents’ social anxiety; therefore improving these constructs may be helpful for adolescents who are at risk of, or are suffering from, higher symptoms of social anxiety. While the use of a cross-sectional design limits ascertainment of direction of association (i.e. it is possible that mindfulness strategies may change after the symptoms of social anxiety disappear), strengthened mindfulness strategies may offer advantages that could plausibly reduce the symptoms of social anxiety. Further research is recommended that may be helpful in establishing the direction of the association. Moreover, these results suggest that pilot interventions designed to strengthen emotion regulation and mindfulness among school adolescents may be used to explore its effectiveness in preventing and managing the symptoms of social anxiety. Examples of such interventions could be; training school teachers to identify social anxiety among students or recruiting a psychological counselor; emphasizing on group learning activities so that fear of talking with people could be minimized. Furthermore, introducing emotion regulation strategies as a part of extracurricular activities in school could be promoted to explore its linkage with stress coping mechanism and social anxiety. An awareness program for teachers and parents aimed at making them aware of and able to assess adolescents’ relationships with their classmates, close friends, teachers, parents, neighbors, and relatives, may help adolescents strengthen their relationships and create favorable environment for adolescents to seek social support to act in adverse conditions.

In the Nepalese setting, this study is a first step towards understanding social anxiety symptoms in the adolescent population. This study has brought forth important findings that may contribute to improving the health of Nepalese adolescents. There are, however, some limitations that must be considered. First, the design of the study being cross-sectional in nature and conducted among non-diagnosed participants, limits the implications of the present research, it does however suggest that emotion dysregulation and dispositional mindfulness and social support may play a part to aggravate symptoms of social anxiety. Second, all of the outcome measures were assessed on the basis of self-reported responses, which could inflate associations. Third, mindfulness could change by training on meditation and previous exposure [9; 30], which has not been assessed in this study. Further, the study sample was drawn from only four secondary schools of Birgunj, which could limit the generalisability of the results in different settings. Finally, multiple statistical tests were performed without adjustment. While interpreting the results, this should be considered, especially given that the association of emotion dysregulation (goal difficulties, impulse and limited strategies), dispositional mindfulness (observing and nonjudge) and social support source (classmates, parents and teachers) with social anxiety symptoms in the multiple regression models was small. Future researches including longitudinal studies should use a larger and diverse study participants utilizing a lab-based methods and using measures of emotion dysregulation and mindfulness specific for adolescents. Furthermore, future studies should also collect data on prior training and exposure to mindfulness activities and emotion regulation strategies.

Despite the limitations of the study, the results indicated that three traits of emotion dysregulation (non-acceptance of emotions, lack of clarity and lack of awareness) were associated with higher symptoms of social anxiety among adolescents, while three traits of dispositional mindfulness (increased acting with awareness, not reacting to inner experience, and being able to describe inner experience) were associated with lower symptoms. Further, strong support from close friends was associated with lower symptoms of social anxiety among adolescents. The current study has produced novel results among Nepalese adolescents, with results that are a first step towards finding a way to reduce the incidence of social anxiety for this population. Although this study shows that gender differences exist with regard to social anxiety with girls reporting higher symptoms, further studies and integration of findings with existing theories is necessary to enhance the understanding of gender differences in social anxiety, thus facilitating gender-sensitive and specifically-tailored policy implementation for both boys and girls with social anxiety. The results indicate the need to consider appropriate counseling and intervention programs that could potentially be used to manage or prevent the undesirable outcomes associated with social anxiety symptoms for adolescents. Future research should focus on more targeted, longitudinal studies, and other methods including case control studies and trials into specific interventions to see if they are useful for increasing emotion dysregulation and dispositional mindfulness in this vulnerable population.
